# Supplementary material for: Effects of brief mindfulness intervention on mental fatigue and recovery in basketball tactical performance
Source: PLoS One. 2024 Dec 31;19(12):e0306815. doi: 10.1371/journal.pone.0306815 (PMC11687761; doi:10.1371/journal.pone.0306815)
Supplement: S1 Appendix — (PDF) [file pone.0306815.s001.pdf]

S1 Appendix. Mindfulness intervention.

1. Centering exercise

This exercise will help you focus on the present moment. This is where you begin the process of learning and mastering the techniques of mindful attention. This exercise will take you 5 minutes to complete. Before engaging in any exercises or activities, remember that success requires the development of specific skills, and investing in the practice of developing those skills is the first step to success.

Please find a sitting position that you think is most comfortable, pay attention to the position of your feet, arms and hands, and gently close your eyes. (Pause for 10 seconds), gently inhale and exhale, and gradually deepen your breath, after a few times in a row, pay attention to the sound and feel of your breath. (Pause 10 seconds).

Now, pay attention to your surroundings. Pay attention to any sounds that may come up. What's inside? What are the sounds outside? (10 second pause) Now focus your attention on how your body feels when you sit in contact with the chair. Pay attention to the physical sensation that occurs in this touch. (10 second pause) Focus on where your hands are on your lap. (10 second pause) Now pay attention to any sensations that other parts of your body may be having, and notice how they change over time. (10 second pause) Don't try to change the feeling, just notice them happening. (10 second pause).

Now, let your mind focus on "Why did you choose me to add this exercise". (10 second pause) Do you have any doubts or other thoughts, just notice them and let them parade through in your mind (10 second pause) and see if you can pay attention to them and acknowledge their existence. (10 second pause) Don't try to dispel them or change them. (10 second pause) Now allow yourself to focus on the performance you want. What is most important to you? What would you like to do with these skills you learned from mindfulness training? (10 second pause).

Stay in a comfortable position for a few seconds, then allow yourself to refocus on the sounds and activities around you. (10 second pause) Focus on your breathing again. (10 second pause) When you are ready, open your eyes and feel that you are focused.

2. Mindful breathing exercise

This short exercise will help expand your mindfulness skills and further develop your mindfulness and mindfulness. The whole exercise takes about 10 minutes. We recommend that you slow down the pace as much as possible.

Please choose a position you think is comfortable to sit, pay attention to your body position, especially the position of your hands, legs and feet, and slowly close your eyes.

(pause for 10 seconds)

Take a few deep breaths and notice and feel the air moving in and out of your body freely. As you inhale, notice the sound of breathing in and feel your own breath (pause for a moment), and similarly, notice the sound of breathing out and feel your breath as you breathe out. Focus your attention on the rising and falling of your belly with each breath. (Pause 10 seconds)

As you continue to breathe, imagine that you have a pen in your hand and draw a line up as you inhale and down as you exhale (pause for 10 seconds). Imagine what the pattern of these lines looks like.

As you continue to breathe slowly, you will notice that various emotions and thoughts may arise in your consciousness, all you have to do is become aware of them because they are an integral part of your consciousness and Allow them to be there, and then again focus your attention on the breath and the various sensations it produces. (10 second pause) Having a variety of emotions and thoughts is simply an objective reflection of human consciousness. There is no need to try to change or control these inner experiences, simply notice the presence of these consciousnesses and refocus your attention on your breath. (pause for 10 seconds)

Continue to inhale and exhale slowly and gently, noticing your thoughts on how your body feels with each breath. When you're ready, slowly open your eyes, be fully aware of your surroundings, and begin what you're going to do next.

3. Body can

The body scan exercise is a deep exploration of the current physical experience. Its goal is to focus on the sensations in the body, to bring mindfulness into the body by being aware and identifying with any feeling or feeling in your body. During a body scan, you focus on the body in a certain order, starting at the feet and ending at the top of the hand. You may notice various bodily sensations: itching, pain, ringing in your ears, lightness, heaviness, heat, cold, etc., and perhaps some neutral sensations. These feelings may be accompanied by certain thoughts or emotions. The awareness of bodily sensations arises only through the identification of bodily sensations. This is very different from thinking about your body. At this point, there is no need to analyze or manipulate your body, just feel and confirm all your current sensations. Record any changes in your mind, mood, or body the first time you do this exercise.

Please do this exercise in a relaxed, distraction-free environment. We recommend lying down for the body scan, but if you find yourself drowsy, or prefer to sit or stand, that's fine. This exercise takes about 15 minutes to complete.

When you're ready, slowly turn your attention to your breath. Now start paying attention

to your breathing, breathing naturally, and paying attention to the tip of your nose and belly. Inhale and realize that you are inhaling; exhale and realize that you are exhaling. Sometimes the mind may move away from this conscious breathing, and when you recognize this, identify with the thoughts that come to your mind, and then return to the focus on the breath, consciously inhaling and exhaling.

Now, gradually move your awareness out of mindful breathing and prepare for a body scan. As you scan your body, you may come across some tense areas. If you can get them to relax, let them relax; if you can't, let the feeling take its course and let it spread where they're going. This can be applied to physical sensations as well as any kind of emotion. As you scan your body, focus on the sensations in your body and any thoughts or emotions that may be triggered by those sensations.

Shift your awareness to an area of your left foot where you can touch the floor. It can be the back heel or the bottom of the left foot. Feel what you feel, feel the heel, big toe, and the sole of your left foot. Feel the top of your toes and left foot, the Achilles below and the ankle above. Now shift your awareness to the lower part of your left leg and feel the calf and calf, and feel where they connect to the knee of your left leg.Raise awareness to the thigh and feel the thigh and its connection to the left arm.

Now withdraw the awareness from the left hip to the left foot, then transfer it to the right foot, bringing awareness to the point where your right foot touches the floor, which can be the back heel or the bottom of the right foot. Feel what you perceive. Feel the heel, big toe, and bottom of your right foot. Feel the top of your toes and right foot, the Achilles below and the ankle above. Now shift your awareness to the lower part of your right leg and feel the calf and calf part and feel where they connect with the knee of your right leg. Raise awareness to the thighs and feel the thighs and where they connect to the right buttocks. Slowly shift your awareness from your right hip to the pelvic area. Pay attention to all feelings, thoughts, and emotions.

Now shift your awareness into the abdomen, which is the site of digestion and absorption, and consciously feel the internal organs and let them take their course. Now shift your awareness from the abdomen to the tailbone, and the awareness begins to move into the lower, middle, and upper back. To feel what you are aware of. Let any tension relax, and if you can't let it go. Now move awareness to the chest, to the heart and lungs. Feel it into the ribs and sternum, then into the breasts. Now slowly withdraw consciousness from the chest and transfer consciousness to the fingertips of the left hand. Feel into the fingers and palm, then the back of the hand, and up to the left wrist.

Continue into the forearm, elbow, upper left arm, and feel what you are aware of. Now move your awareness to the fingertips of your right hand. Feel into the fingers and palm, then the back of the hand, and rise to the bowl of the right hand. Continue into your forearm, arm, upper right arm, and feel what you are aware of. Bring awareness into both shoulders and armpits, then up into the neck and throat. Experience all the feelings,

thoughts and emotions. Now move your awareness into the jaw, then slowly to the teeth, tongue, mouth, lips. Let the senses go wherever they need to go and leave them alone. Feelings go into the face, the sinus tunnels deep in the head, the eyes, the muscles around the eyes. Sensation enters the forehead and temporal lobes. for a while.

Allow awareness to enter the top of the head and the back of the head. The sensation goes into the ear, then into the head, and into the brain. for a while. Now, from head to toe, expand awareness to the entire body. Connect the head, neck, shoulders, arms, hands, chest, back, abdomen, buttocks, pelvic area, legs, and feet. Feel the body as a whole organism, with its physical sensations, thoughts, and emotions. for a while. Inhale and feel your entire body lift; continue to inhale deeply, then exhale while feeling your body descend. Feel the body as a whole. After a while, end this body scan exercise.
