# Supplementary material for: Effects of brief mindfulness intervention on mental fatigue and recovery in basketball tactical performance
Source: PLoS One. 2024 Dec 31;19(12):e0306815. doi: 10.1371/journal.pone.0306815 (PMC11687761; doi:10.1371/journal.pone.0306815)
Supplement: Attachment — Submitted filename: Response to Reviewers.docx [file pone.0306815.s002.docx]

**Dear reviewer 1,**

Thank you very much for your attention, evaluation and comments on our paper. We revised the manuscript in accordance with your kind and detailed comments, and carefully proof-read the manuscript to minimize typographical, grammatical, and bibliographical errors. We made many revisions according to your comments. A document answering every question from you was summarized and enclosed. And a revised manuscript with the correction sections yellow marked was attached as the supplemental material and for easy check/editing purpose.

**Introduction**

1. **The new aspects of the study compared to previous studies should be highlighted in more detail.**

**Line 73-79. Line 90-94. Line 97-103**. According to the advice, more detailed introduction was added.

**Material and method**

2. **It is well structured and detailed.**

Thanks.

**Results**

3. **They are well interpreted, and the tables are easy to view.**

Thanks.

**Discussions**

4. **Lines 344-346 need references**

**Line 366.** The reference was added.

5. **To explain the practical implications of the most relevant results of the study on the training of basketball players.**

**Line 418-424.** According to this suggestion, some practical implications were added.

**Conclusions.**

6. **Future research directions to be added.**

**Line 441-446.** The future directions were added here.

Finally, we are very grateful to the reviewer for the detailed and kind suggestions. We are deeply inspired. We hope that the reviewer will be satisfied with our revisions and our revised version will be satisfactory for publication in PLOS ONE. Please do not hesitate to contact us if you have any new suggestions. Great thanks to you.

Best regards

Sincerely yours

**Dear reviewer 2,**

Thank you very much for your attention, evaluation and comments on our paper. We made many revisions in accordance with your kind and detailed comments, and carefully proof-read the manuscript to minimize typographical, grammatical, and bibliographical errors. A document answering every question from you was summarized and enclosed. And a revised manuscript with the correction sections red marked was attached as the supplemental material and for easy check/editing purpose.

**Introduction:**

1. **Lack of references to original research: the introduction relies quite heavily on review articles rather than direct empirical studies, which could diminish the empirical foundation of your research. I recommend including specific original research studies for each mentioned effect of mental fatigue.**

**Line 51-55. Line 73-79. Line 90-94. Line 97-103**. According to this suggestion, some empirical references were added in this part.

2. **Point of insertion for the discussion on reaction time: currently, there is an absence of discussion on the impact of mental fatigue on reaction times, an essential factor in sports like basketball. It would be beneficial to add a section that discusses previous findings on reaction times under mental fatigue.**

**Line 90-94.** The effect of mental fatigue on reaction time was explained here.

**Methodology:**

3. **Clarity and justification of the Stroop Task: the justification for using the Stroop Task to induce mental fatigue lacks detailed explanation. I advise strengthening this section by citing studies that have utilized similar methodologies.**

**Line 223-227.** More description of Stroop task was introduced, and many related references were also added here.

4. **Data collection and protocol: the manuscript would benefit from more detailed descriptions of the data collection methods and experimental protocols. Enhancing the description of the experimental setup and data collection procedures would be highly advantageous.**

**Line 259-264.** More details of the experiment were added here. And the information about the experimental protocols were added in the Appendix. Thank you very much.

**Results:**

5. **Presentation and clarity of data: the presentation of the results could be clearer. I advise reorganizing this section to include more detailed tables and clearer figures.**

All the related tables and figures were provided in this manuscript. Because we have many tables and figures, we make them together for the readers to see. And we have bolded all the symbols in the figures for readers to see it clearly. If you still think we lack some tables or figures, please tell us without any hesitate, we will improve it. Thank you very much.

**Discussion and Conclusion:**

6. **Alignment of the discussion with the results: it is crucial to ensure that the discussion remains closely aligned with the empirical findings. I suggest revising this section to avoid any overgeneralization not directly supported by the results.**

According to the advice, we deleted some unrelated sentences and also added some related sentences according to your and another reviewer’s advice (e.g. Line 418-424).

7. **Consistency and concluding remarks: the conclusion might not effectively encapsulate the research findings. I recommend providing a more concise summary of the findings in the conclusion.**

**Line 441-446**. According to the advice, we revised this part.

Finally, we are very grateful to the reviewer for the detailed and kind suggestions. We are deeply inspired. We hope that the reviewer will be satisfied with our revisions and our revised version will be satisfactory for publication in PLOS ONE. And please do not hesitate to contact us if you have any new suggestion. Great thanks to you.

Best regards

Sincerely yours
